# The robustness of porin-cytochrome gene clusters from *Geobacter metallireducens* in extracellular electron transfer

**DOI:** 10.1128/mbio.00580-24

**Published:** 2024-08-02

**Authors:** Shiyan Zhuo, Yongguang Jiang, Lei Qi, Yidan Hu, Zhou Jiang, Yiran Dong, Liang Shi

**Affiliations:** 1 Department of Biological Sciences and Technology, School of Environmental Studies, China University of Geosciences, Wuhan, China; 2 State Key Laboratory of Microbial Resources, Institute of Microbiology, Chinese Academy of Sciences, Beijing, China; 3 State Key Laboratory of Biogeology and Environmental Geology, China University of Geosciences, Wuhan, China; 4 State Environmental Protection Key Laboratory of Source Apportionment and Control of Aquatic Pollution, Ministry of Ecology and Environment, China University of Geosciences, Wuhan, China; 5 Hubei Key Laboratory of Yangtze Catchment Environmental Aquatic Science, China University of Geosciences, Wuhan, China; California Institute of Technology, Pasadena, California, USA; University of Minnesota, St. Paul, Minnesota, USA

**Keywords:** porin-cytochrome gene clusters, *Geobacter metallireducens*, extracellular reduction of Fe(III), extracellular reduction of anodes, direct interspecies electron transfer

## Abstract

To investigate their roles in extracellular electron transfer (EET), the porin-cytochrome (*pcc*) gene clusters *Gmet0825-0828*, *Gmet0908-0910*, and *Gmet0911-0913* of the Gram-negative bacterium *Geobacter metallireducens* were deleted. Failure to delete all *pcc* gene clusters at the same time suggested their essential roles in extracellular reduction of Fe(III)-citrate by *G. metallireducens*. Deletion of *Gmet0825-0828* had no impact on bacterial reduction of Fe(III)-citrate but diminished bacterial reduction of ferrihydrite and abolished anode reduction and direct interspecies electron transfer (DIET) to *Methanosarcina barkeri* and *Geobacter sulfurreducens*. Although it had no impact on the bacterial reduction of Fe(III)-citrate, deletion of *Gmet0908-0910* delayed ferrihydrite reduction, abolished anode reduction, and diminished DIET. Deletion of *Gmet0911-0913* had little impact on DIET but diminished bacterial reductions of Fe(III)-citrate, ferrihydrite, and anodes. Most importantly, deletions of both *Gmet0825-0828* and *Gmet0908-0910* restored bacterial reduction of ferrihydrite and anodes and DIET. Enhanced expression of *Gmet0911-0913* in this double mutant when grown in coculture with *G. sulfurreducens* Δ*hybL*Δ*fdnG* suggested that this cluster might compensate for impaired EET functions of deleting *Gmet0825-0828* and *Gmet0908-0910*. Thus, these *pcc* gene clusters played essential, distinct, overlapping, and compensatory roles in EET of *G. metallireducens* that are difficult to characterize as deletion of some clusters affected expression of others. The robustness of these *pcc* gene clusters enabled *G. metallireducens* to mediate EET to different acceptors for anaerobic growth even when two of its three *pcc* gene clusters were inactivated by mutation. The results from this investigation provide new insights into the roles of *pcc* gene clusters in bacterial EET.

**IMPORTANCE:**

The Gram-negative bacterium *Geobacter metallireducens* is of environmental and biotechnological significance. Crucial to the unique physiology of *G. metallireducens* is its extracellular electron transfer (EET) capability. This investigation sheds new light on the robust roles of the three porin-cytochrome (*pcc*) gene clusters, which are directly involved in EET across the bacterial outer membrane, in the EET of *G. metallireducens*. In addition to their essential roles, these gene clusters also play distinct, overlapping, and compensatory roles in the EET of *G. metallireducens*. The distinct roles of the *pcc* gene clusters enable *G. metallireducens* to mediate EET to a diverse group of electron acceptors for anaerobic respirations. The overlapping and compensatory roles of the *pcc* gene clusters enable *G. metallireducens* to maintain and restore its EET capability for anaerobic growth when one or two of its three *pcc* gene clusters are deleted from the genome.

## INTRODUCTION

The Gram-negative bacterium *Geobacter metallireducens* is among the first microorganisms experimentally demonstrated to be capable of respiring on insoluble metal oxides for anaerobic growth via extracellular electron transfer (EET) (1–4). In addition to metal oxides, *G. metallireducens* can also mediate EET with electrodes (5), stainless steel (6), and microbial cells of other species (7–11). The latter is also referred to as direct interspecies electron transfer (DIET). In DIET, *G. metallireducens* oxidizes ethanol and then transfers the released electrons directly to the bacterium *Geobacter sulfurreducens* (7) or the archaea, such as *Methanosarcina barkeri*, *Methanosarcina acetivorans* or *Methanosarcina subterranea* (8, 10), *Methanosaeta harundinacea* (9), and *Methanothrix thermoacetophila* (11). *G. sulfurreducens* uses the received electrons to reduce fumarate to succinate (7), while the archaea use the electrons to produce methane (8–11).

As a Gram-negative bacterium, *G. metallireducens* possesses the outer membrane that is electrically non-conductive (3, 12). To transfer electron across the outer membrane, Gram-negative bacteria use the protein complexes that consist of outer membrane porin proteins and redox proteins (13–21). The porin proteins function as the sheaths to insulate the redox proteins from the outer membrane. Inside the porin proteins, the redox proteins transfer electrons across the outer membrane (13, 14, 16–20). These protein complexes are the key components of the EET pathways for a variety of Gram-negative bacteria (14, 22, 23).

**Fig 1:**
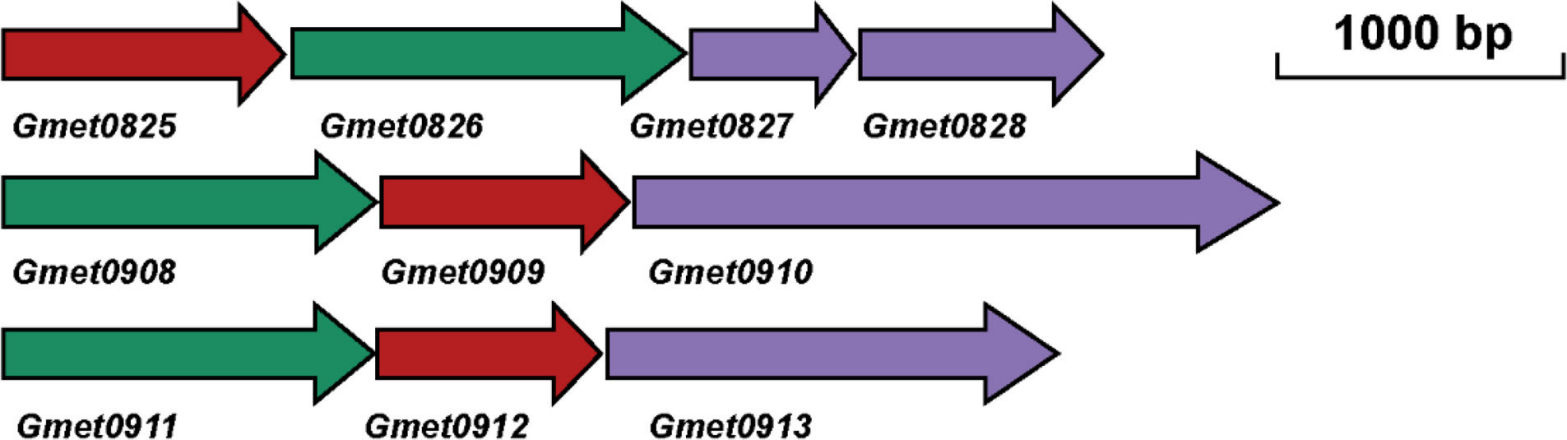
The *pcc* gene clusters of *Geobacter metallireducens*. Green, the genes of the putative outer membrane porin proteins; red, the genes of the putative periplasmic *c*-type cytochromes (*c*-Cyts); purple, the genes of the putative outer membrane *c*-Cyts. The putative periplasmic *c*-Cyts Gmet0825, Gmet0909, and Gmet0912 contain 12, 9, and 8 heme-binding motifs, respectively. The putative outer membrane porin proteins Gmet0826, Gmet0908, and Gmet0911 are all predicted to contain 20 *trans* outer membrane motifs. The putative outer membrane *c*-Cyts Gmet0827, Gmet0828, Gmet0910, and Gmet0913 possess 5, 6, 10, and 9 heme-binding motifs, respectively. The mutants made in this investigation including Δ1, deletion of *Gmet0825-2828*; Δ2, deletion of *Gmet0908-0910*; Δ3, deletion of *Gmet0911-0913*; Δ4, deletion of both *Gmet0825-2828* and *Gmet0908-0910;* and Δ5, deletion of *pilA-N* (not shown here). For mutant details, please see Table S1.

Among those in *Geobacter* species, the porin-cytochrome protein complexes (Pcc) of *G. sulfurreducens* are the most well characterized, in which they are essential for EET across the outer membrane to the metal oxides, anodes, and bacterium *Prosthecochloris aestaurii* (17, 24–27). The genes for Pcc of *G. sulfurreducens* are adjacent to each other to form gene clusters (15, 17). The porin-cytochrome (*pc*c) gene clusters are also found in many *Geobacter* and other bacterial species (15). In *G. metallireducens*, three *pcc* gene clusters are identified and they are *Gmet0825-0828* (*pccH*), *Gmet0908-0910* (*pccF*), and *Gmet0911-0913* (*pccG*) (10, 15) (Fig. 1). It should be noted that *Gmet0908-0913* clusters differed from *ombBomaBomcB-ombComaComcC* clusters of *G. sulfurreducens*. In *G. sulfurreducens*, OmbBOmaBOmcB were 70%–100% identical to OmbCOmaCOmcC. In *G. metallireducens*, Gmet0908-0910 were only 26%–62% identical to Gmet0911-0913 and 26%–61% identical to OmbBOmaBOmcB/OmbCOmaCOmcC. Gmet0911-0913 were 30%–75% identical to OmbBOmaBOmcB/OmbCOmaCOmcC. The *c*-Cyts proposed to be inserted to the porin proteins and the porin proteins were more conserved than the *c*-Cyts on the outer membrane surface (15, 17, 25).

Previous results showed that deletions of *Gmet0910*, *Gmet0912*, or *Gmet0913* had little impact on the bacterial ability to reduce Fe(III)-citrate and Fe(III)-oxides (28). However, deletions of *Gmet0910*, *Gmet0912*, or *Gmet0913* all lowered the ability of mutants to form coculture with *M. barkeri*, *M. acetivorans*, or *M. subterranea* during the initial stage of coculture. These mutants then regained their ability to form cocultures with these archaea in the late stage of coculturing. Deletions of *Gmet0910* or *Gmet0913* also lowered the bacterial ability to form coculture with *G. sulfurreducens* (10). Further investigation demonstrated the increased mRNA abundance of *Gmet0908-0910* when cocultured with *M. thermoacetophila*, suggesting their importance in DIET from *G. metallireducens* to *M. thermoacetophila* (11).

Despite above advances, the roles of Gmet0825-0828 in EET have never been experimentally investigated. Moreover, the contributing roles of these complexes in EET have never been systematically compared. To these ends, the gene clusters *Gmet0825-0828*, *Gmet0908-0910*, and *Gmet0911-0913* were each deleted and a double mutant without *Gmet0825-0828* and *Gmet0908-0910* (Δ*Gmet0825-0828*Δ*Gmet0908-0910*) was also constructed. Previous results showed the deletion or modification of the *pilA-N* gene for *G. metallireducens* impaired bacterial EET ability (8, 29, 30). Thus, the *pilA-N* gene of *G. metallireducens* was also deleted and Δ*pilA-N* served as a control. The resulting mutants were tested for their ability to reduce Fe(III)-citrate, ferrihydrite, and anodes as well as to form cocultures with *M. barkeri* and the wild type (WT) and Δ*hybL*Δ*fdnG* of *G. sulfurreducens*. Results showed that the three *pcc* gene clusters of *G. metallireducens* could not be deleted at the same time, suggesting their unknown essential roles or the regulatory effects of these clusters in anaerobic growth of *G. metallireducens* unrelated to EET. The gene-cluster-deletion mutants also displayed varied abilities to reduce Fe(III)-citrate, ferrihydrite, and anodes and to form cocultures with *M. barkeri* and *G. sulfurreducens*, demonstrating that the Pcc complexes contributed to the EET of *G. metallireducens* differently. The involvement of *Gmet0825-0828*, *Gmet0908-0910*, and *Gmet0911-0913* in ferrihydrite reduction further suggested their overlapping roles in ferrihydrite reduction. Most importantly, the findings that the mutant without either *Gmet0825-0828* or *Gmet0908-0910* regained its EET ability and that deletion of these clusters increased expression of *Gmet0911-0913* provided new evidence for regulatory complexity in these *pcc* gene clusters.

## RESULTS

### Fe(III) reductions

To compare their roles in EET, four gene-cluster-deletion mutants were made. They were three single-gene-cluster-deletion mutants Δ*Gmet0825-0828*, Δ*Gmet0908-0910*, and Δ*Gmet0911-0913* and a double-gene-cluster-deletion mutant Δ*Gmet0825-0828*Δ*Gmet0908-0910* (Table S1). Despite several attempts with Δ*Gmet0825-0828*Δ*Gmet0908-0910*, the triple-gene-cluster-deletion mutant Δ*Gmet0825-0828*Δ*Gmet0908-0910*Δ*Gmet0911-0913* could not be constructed under the conditions tested. The Δ*pilA-N* was prepared as a control (Table S1).

**Fig 2:**
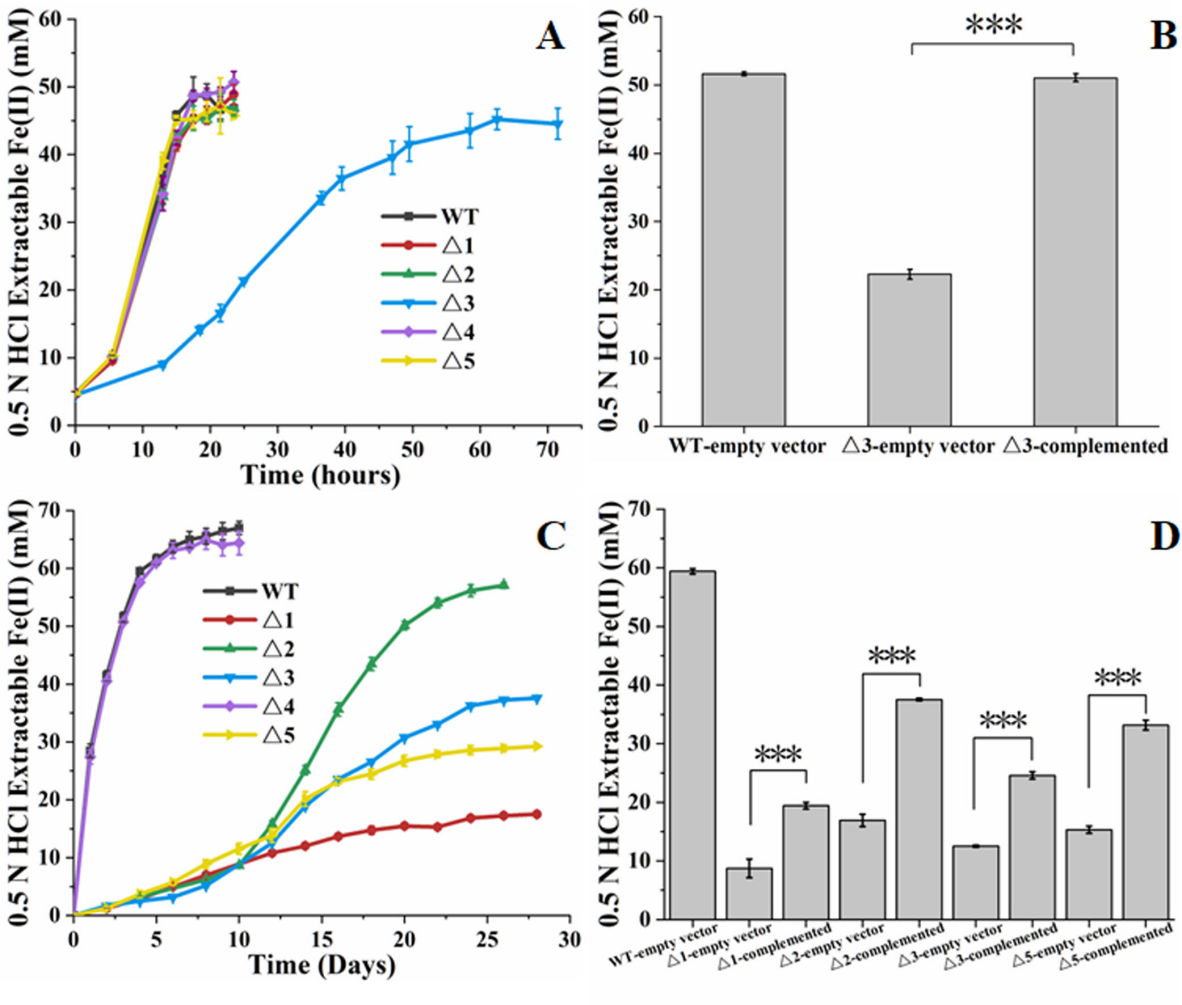
Fe(III) reduction by *Geobacter metallireducens* and its mutants. (**A**) Fe(III)-citrate reduction over 24 to 72 hours. (**B**) Fe(III)-reduction at 24 hours after inoculation. (**C**) Ferrihydrite reduction over 10 to 26 days. (**D**) Ferrihydrite reduction at 10 days after inoculation. All results are reported as mean and standard error of the mean (*n* = 3). For points with no error bar, the error was smaller than the size of the symbol. In panels B and D, Student’s *t* test was used for comparing the mutant complemented and those with empty vector. ***P ≤ 0.001. WT, wild type of *G. metallireducens*; Δ1, Δ*Gmet0825-0828*; Δ2, Δ*Gmet0908-0910* Δ3, Δ*Gmet0911-0913*; Δ4, Δ*Gmet0825-0828*Δ*Gmet0908-0910*; Δ5, Δ*pilA-N*.

The constructed mutants were compared for their ability to reduce Fe(III)-citrate and ferrihydrite with that of the WT of *G. metallireducens*. Except for Δ*Gmet0911-0913*, all other mutants showed the similar ability of reducing Fe(III)-citrate to that of WT. Δ*Gmet0911-0913* displayed delayed ability to reduce Fe(III)*-*citrate, as compared with that of WT and other mutants of *G. metallireducens* (Fig. 2A). Compared with that with empty vector, the ability of Δ*Gmet0911-0913* complemented with cloned *Gmet0911-0913* for Fe(III)-citrate reduction was significantly increased (Fig. 2B).

Except for Δ*Gmet0825-0828*Δ*Gmet0908-0910*, all other mutants showed diminished ability to reduce ferrihydrite, as compared with that of the WT of *G. metallireducens* (Fig. 2C). At 10 days after inoculation, reductions of ferrihydrite by WT and Δ*Gmet0825-0828*Δ*Gmet0908-0910* plateaued, while other mutants reduced ferrihydrite at much slower rates. After that, Δ*Gmet0825-0828*, Δ*Gmet0908-0910*, Δ*Gmet0911-0913*, and Δ*pilA-N* reduced ferrihydrite at different rates. At 26 days after inoculations, the ability to reduce ferrihydrite decreased in the order of Δ*Gmet0908-0910* > Δ*Gmet0911-0913* > Δ*pilA-N* > Δ*Gmet0825-0828* (Fig. 2C). Compared with those with empty vector, all single-gene-cluster-deletion mutants complemented with their respective gene cluster showed some increased ability to reduce ferrihydrite (between 33% and 63% of WT) but in no case was complementation able to recover WT reduction (Fig. 2D).

### Anode reduction

**Fig 3:**
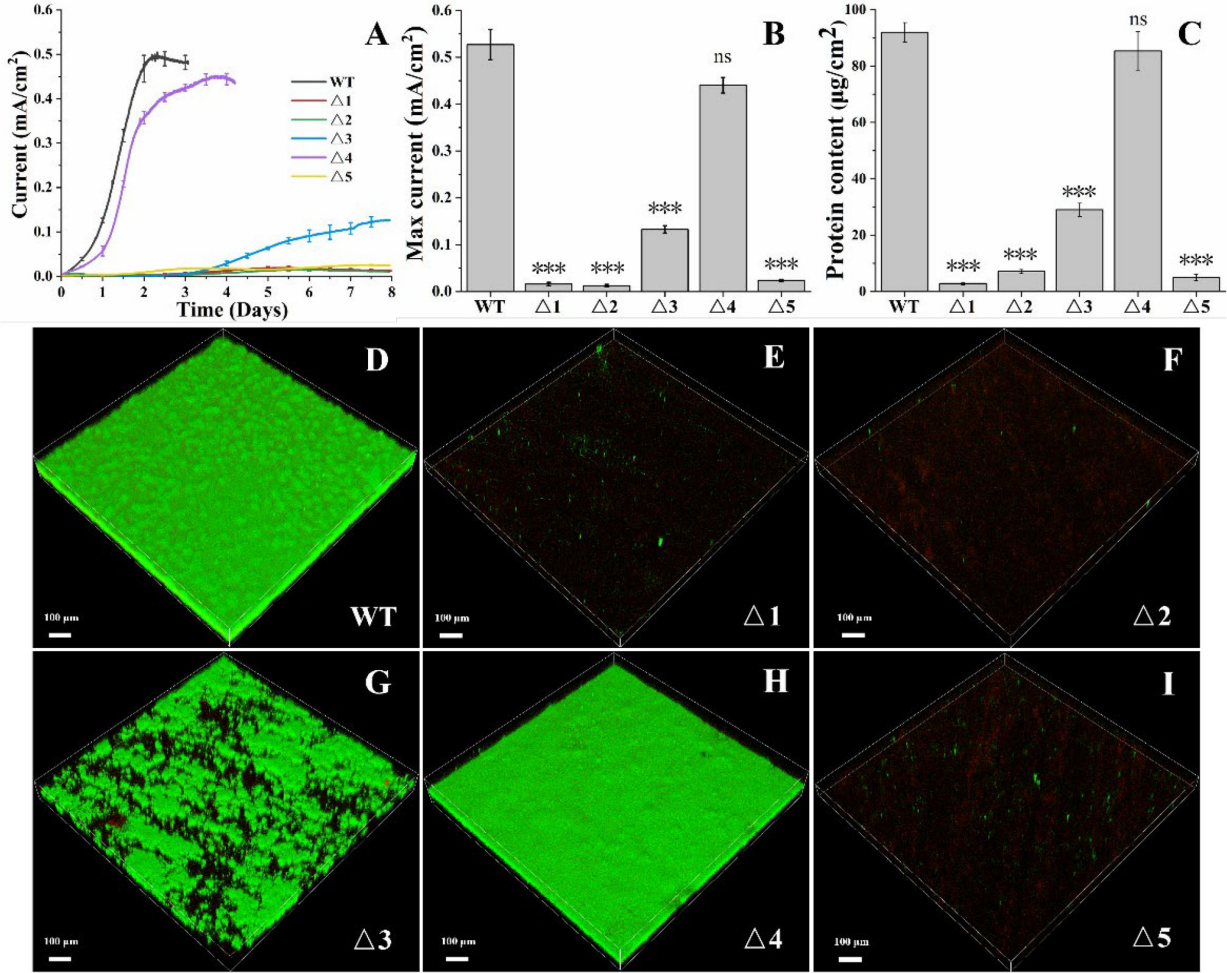
Anode reduction by *Geobacter metallireducens* and its mutants. (**A**) Current production. (**B**) Maximum current generated. (**C**) Bacterial protein content on anodes. All results are reported as mean and standard error of the mean (*n* = 3). For points with no error bar, the error was smaller than the size of the symbol. (**D–I**) Biofilms on anodes of different bacterial strains. In panels B and C, Student’s *t* test was used for comparing WT and the mutants. ns, *P* > 0.05 and ***P ≤ 0.001. WT, wild type of *G. metallireducens*; Δ1, Δ*Gmet0825-0828*; Δ2, Δ*Gmet0908-0910*; Δ3, Δ*Gmet0911-0913*; Δ4, Δ*Gmet0825-0828*Δ*Gmet0908-0910*; Δ5, Δ*pilA-N*.

The electricity production by the WT of *G. metallireducens* was detected at 1 day after growth and plateaued at 2 days after growth (Fig. 3A). Compared with the WT, Δ*Gmet0825-0828*Δ*Gmet0908-0910* showed a delayed ability to produce electricity on anodes. The electricity production of Δ*Gmet0825-0828*Δ*Gmet0908-0910* plateaued at 3 days after growth (Fig. 3A). Little electricity was produced by Δ*Gmet0911-0913* during the first 3 days of growth. The electricity production by Δ*Gmet0911-0913* then increased (Fig. 3A). During 8 days of growth, little electricity was produced by Δ*Gmet0825-0828*, Δ*Gmet0908-0910*, or Δ*pilA-N* (Fig. 3A). The maximum electricity production detected decreased in the order of WT *≈* Δ*Gmet0825-0828*Δ*Gmet0908-0910 >>* Δ*Gmet0911-0913* >> Δ*Gmet0825-0828 ≈* Δ*Gmet0908-0910 ≈* Δ*pilA-N* (Fig. 3B). The amounts of protein and biofilm detected on the electrode surface also decreased in the same order (Fig. 3C through I).

### Coculture with *M. barkeri*

**Fig 4:**
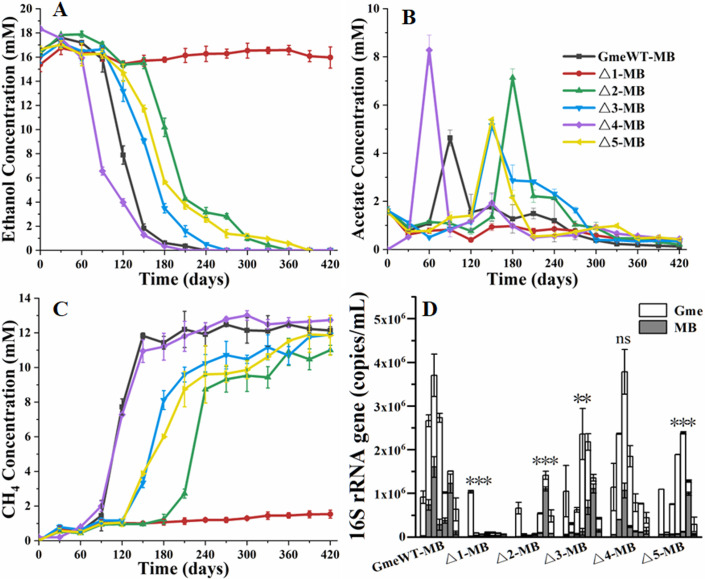
The first generation of cocultures between WT and gene-deletion mutants of *Geobacter metallireducens* (Gme) and *Methanosarcina barkeri* (MB). (**A**) Ethanol metabolism. (**B**) Acetate metabolism. (**C**) Methane production. (**D**) The copies of combined microbial 16S rRNA genes. All results are reported as mean and standard error of the mean (*n* = 3). For points with no error bar, the error was smaller than the size of the symbol. In panel **D**, samples were collected at 0, 60, 120, 180, 240, 300, and 360 days after cocultures. Student’s *t* test was used for comparing the maximum copies of combined microbial 16S rRNA genes of different cocultures. ns, *P* > 0.05, **P ≤ 0.01, and ***P ≤ 0.001. WT, wild type of *G. metallireducens*; Δ1, Δ*Gmet0825-0828*; Δ2, Δ*Gmet0908-0910*; Δ3, Δ*Gmet0911-0913*; Δ4, Δ*Gmet0825-0828*Δ*Gmet0908-0910*; Δ5, Δ*pilA-N*.

In the first generation, all other mutants could coculture with *M. barkeri*, except for Δ*Gmet0825-0828* that could not form coculture with *M. barkeri* (Fig. 4A through D). Compared with that of the WT of *G. metallireducens*, the coculture of Δ*Gmet0825-0828*Δ*Gmet0908-0910* oxidized ethanol more quickly (Fig. 4A and B). However, no major difference was detected in methane production (Fig. 4C) and maximum copies of microbial 16S rRNA genes (Fig. 4D) between the coculture with the WT and that of Δ*Gmet0825-0828*Δ*Gmet0908-0910*. Compared with that of the WT and Δ*Gmet0825-0828*Δ*Gmet0908-0910*, the cocultures of Δ*Gmet0908-0910*, Δ*Gmet0911-0913*, or Δ*pilA-N* showed delayed ability to oxidize ethanol and to produce methane (Fig. 4A through C). The maximum copies of microbial 16S rRNA genes detected in the cocultures of Δ*Gmet0911-0913* and Δ*pilA-N* were similar to each other, but lower than that of WT and Δ*Gmet0825-0828*Δ*Gmet0908-0910* and higher than that of Δ*Gmet0908-0910* (Fig. 4D; Table S4).

In the second generation, the cocultures with WT and Δ*Gmet0825-0828*Δ*Gmet0908-0910* displayed no difference in terms of their oxidation of ethanol and formation of methane (Fig. S1A through C). However, the maximum copies of 16S rRNA genes detected in the coculture with the WT was lower than that with Δ*Gmet0825-0828*Δ*Gmet0908-0910* (Fig. S1D). The ability of the coculture with Δ*Gmet0911-0913* to oxidize ethanol and to produce methane was lower than that with WT and Δ*Gmet0825-0828*Δ*Gmet0908-0910* but higher than that with Δ*Gmet0908-0910* and Δ*pilA-N* (Fig. S1A through C). Similarly, the maximum copies of microbial 16S RNA genes detected in the cocultures of Δ*Gmet0911-0913* were also lower than those of WT and Δ*Gmet0825-0828*Δ*Gmet0908-0910*, but higher than those of Δ*Gmet0908-0910* and Δ*pilA-N* (Fig. S1D; Table S4). Fluorescence *in situ* hybridization (FISH) analyses of the formed granules confirmed the coculture of *G. metallireducens-M. barkeri* (Fig. S2A and B).

### Coculture with *G. sulfurreducens*

**Fig 5:**
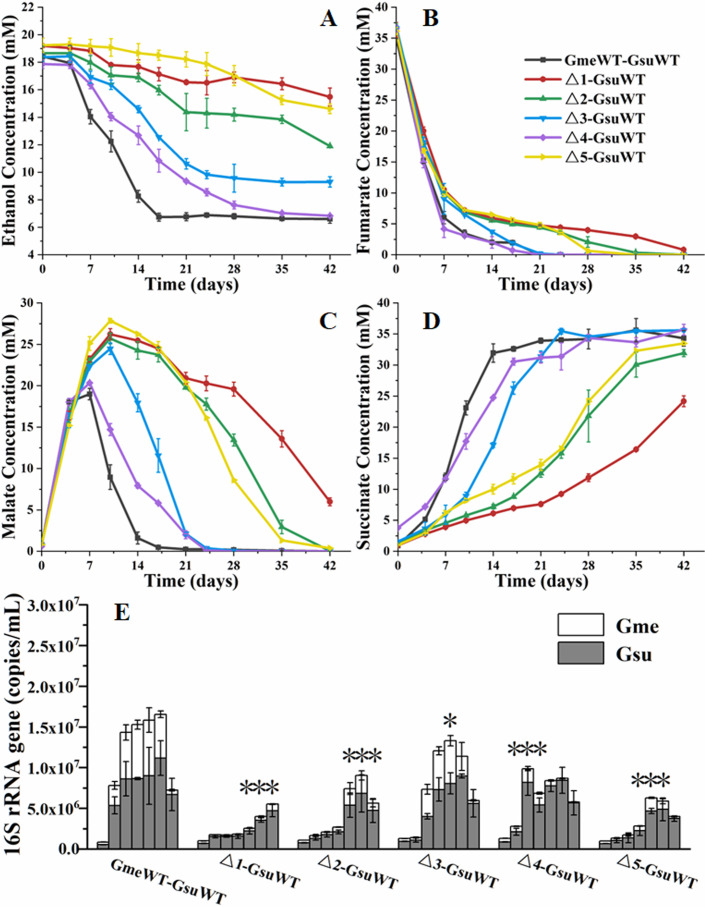
The first generation of cocultures between WT and gene-deletion mutants of Gme and *Geobacter sulfurreducens* (Gsu). (**A**) Ethanol metabolism. (**B**) Fumarate metabolism. (**C**) Malate metabolism. (**D**) Succinate production. (**E**) The copies of combined bacterial 16S rRNA genes. All results are reported as mean and standard error of the mean (*n* = 3). For points with no error bar, the error was smaller than the size of the symbol. In panel **E**, samples were collected at 0, 7, 14, 21, 28, 35, and 42 days after cocultures. Student’s *t* test was used for comparing the maximum copies of combined bacterial 16S rRNA genes of different cocultures. *P ≤ 0.05 and ***P ≤ 0.001. WT, wild type of *G. metallireducens*; Δ1, Δ*Gmet0825-0828*; Δ2, Δ*Gmet0908-0910*; Δ3, Δ*Gmet0911-0913*; Δ4, Δ*Gmet0825-0828*Δ*Gmet0908-0910*; Δ5, Δ*pilA-N*.

In the first generation of coculture, all constructed mutants of *G. metallireducens* could grow with the WT of *G. sulfurreducens* (Fig. 5A through E). However, their abilities to coculture with the WT of *G. sulfurreducens* were reduced, as compared with that of WT *G. metallireducens*. Among the mutants tested, the coculture with Δ*Gmet0825-0828*Δ*Gmet0908-0910* oxidized ethanol (Fig. 5A) and reduced fumarate (Fig. 5B through D) faster than that with Δ*Gmet0825-0828,* Δ*Gmet0908-0910*, Δ*Gmet0911-0913*, and Δ*pilA-N*. Correspondingly, the copies of bacterial 16S rRNA genes in the coculture with Δ*Gmet0825-0828*Δ*Gmet0908-0910* also plateaued faster than that of Δ*Gmet0825-0828*, Δ*Gmet0908-0910*, Δ*Gmet0911-0913*, and Δ*pilA-N* (Fig. 5E). However, maximum copies of bacterial 16S rRNA genes detected decreased in the order of the cocultures with WT > Δ*Gmet0911-0913* > Δ*Gmet0908-0910 ≈* Δ*Gmet0825-0828*Δ*Gmet0908-0910 >* Δ*pilA-N* > Δ*Gmet0825-0828* (Fig. 5E; Table S5).

In the second generation of cocultures, Δ*pilA-N* and Δ*Gmet0825-0828* of *G. metallireducens* could not grow with the WT of *G. sulfurreducens*. The cocultures with the WT, Δ*Gmet0911-0913*, and Δ*Gmet0825-0828*Δ*Gmet0908-0910* were similar in terms of their ethanol oxidation, fumarate reduction, malate metabolism, and maximum copies of bacterial 16S rRNA genes detected (Fig. S3). Compared with that with the WT, Δ*Gmet0911-0913*, and Δ*Gmet0825-0828*Δ*Gmet0908-0910*, the coculture with Δ*Gmet0908-0910* showed a reduced ability to oxidize ethanol and to reduce fumarate (Fig. S3A through D). Similarly, maximum copies of bacterial 16S rRNA genes detected in the coculture with Δ*Gmet0908-0910* were lower than those in the cocultures with the WT, Δ*Gmet0911-0913*, andΔ*Gmet0825-0828*Δ*Gmet0908-0910* (Fig. S3E; Table S5). The coculture of *G. metallireducens-G. sulfurreducens* was also confirmed by FISH analyses (Fig. S2C and D).

Previous results indicated that indirect interspecies electron transfer from *G. metallireducens* to *G. sulfurreducens*, such as that mediated by H_2_, could overshadow the roles of bacterial genes in DIET from *G. metallireducens* to *G. sulfurreducens* (31). To avoid interference from indirect interspecies electron transfer, the *hybL* and *fdnG* genes for *G. sulfurreducens* were deleted, as they were involved in indirect interspecies electron transfer (32). The WT and mutants of *G. metallireducens* were then cocultured with *G. sulfurreducens* Δ*hybL*Δ*fdnG*. Compared with that with the WT of *G. metallireducens* and WT of *G. sulfurreducens*, the coculture with the WT of *G. metallireducens* and *G. sulfurreducens* Δ*hybL*Δ*fdnG* was enhanced (Fig. S4 and S5). Similar to that with the WT of *G. sulfurreducens*, the mutants of *G. metallireducens* could coculture with *G. sulfurreducens* Δ*hybL*Δ*fdnG* in the first generation and their ability to form coculture was lower than that with the WT of *G. metallireducens* (Fig. S4; Table S6). In the second generation of cocultures, WT, Δ*Gmet0911-0913*, and Δ*Gmet0825-0828*Δ*Gmet0908-0910* of *G. metallireducens* grew well with *G. sulfurreducens* Δ*hybL*Δ*fdnG*; Δ*Gmet0908-0910* grew poorly with *G. sulfurreducens* Δ*hybL*Δ*fdnG*; and Δ*pilA-N* and Δ*Gmet0825-0828* could not grow with *G. sulfurreducens* Δ*hybL*Δ*fdnG* (Fig. S5; Table S6). These were similar to those grown with the WT of *G. sulfurreducens* except that Δ*Gmet0908-0910* grew more poorly with *G. sulfurreducens* Δ*hybL*Δ*fdnG* than with WT of *G. sulfurreducens* (Fig. S3; Table S5)

### Transcriptomic and immunoblot analyses

**Fig 6:**
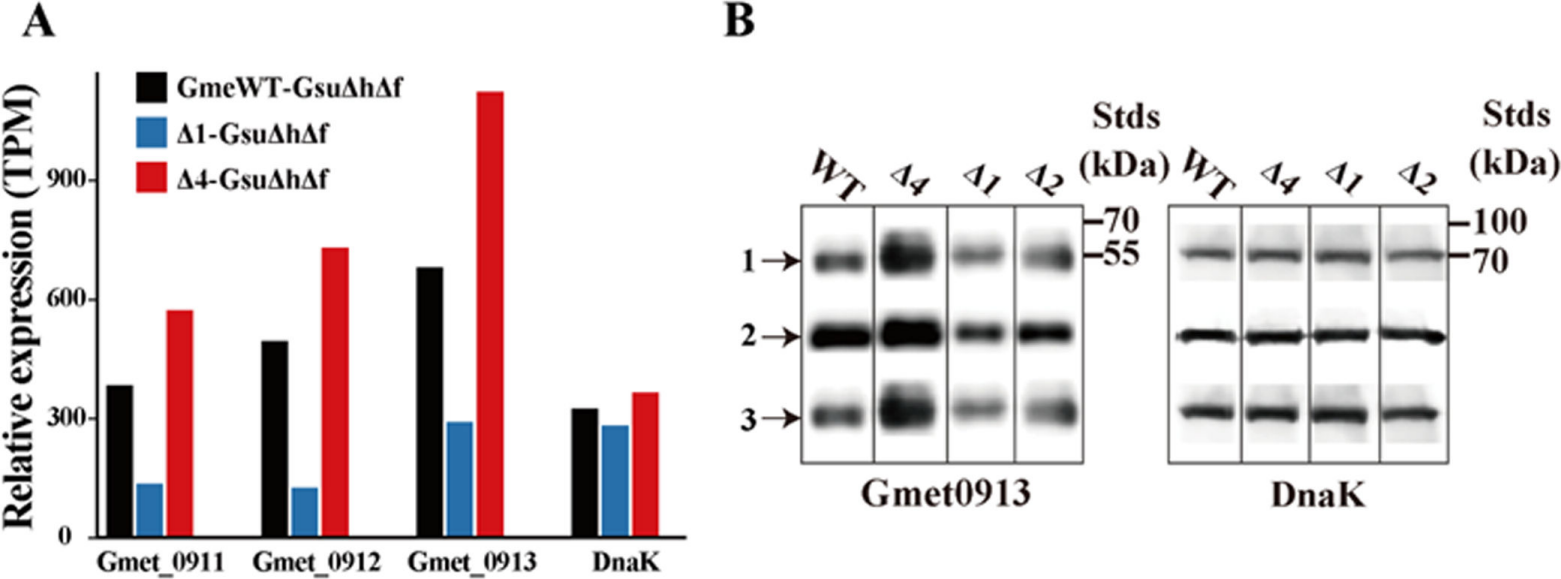
Transcriptomic and immunoblot analyses. (**A**) The relative abundance of *Gmet0911*, *Gmet0912*, *Gmet0913*, and *dnaK* genes in the cocultures between WT and gene-deletion mutants of Gme and *Geobacter sulfurreducens* Δ*hybL*Δ*fdnG* (GsuΔ*h*Δ*f*) at 14 days after coculture. (**B**) Gmet0913 and Dnak detected under different conditions with their respective antibodies. The migration positions of standard proteins (Stds) in kilodaltons (kDa) are shown on the right. 1, samples collected at 24 hours after reduction of Fe(III)-citrate; 2, samples collected at 10 days after reduction of ferrihydrite; 3, samples collected at 14 days after coculture with GsuΔ*h*Δ*f*; Δ1, Δ*Gmet0825-0828;* Δ2, Δ*Gmet0908-0910*; Δ4, Δ*Gmet0825-0828*Δ*Gmet0908-0910*.

To investigate the mechanism underlying the restored ability of Δ*Gmet0825-0828*Δ*Gmet0908-0910* for growth with *G. sulfurreducens* Δ*hybL*Δ*fdnG*, transcriptomes of the cocultures of WT, Δ*Gmet0825-0828*, and Δ*Gmet0825-0828*Δ*Gmet0908-0910* of *G. metallireducens* with *G. sulfurreducens* Δ*hybL*Δ*fdnG* at 14 days of the first generation were compared by RNA sequencing (RNAseq). A total of 3,440 genes of *G. metallireducens* were identified, and principal component analysis showed the distinct transcriptomes among the cocultures of WT, Δ*Gmet0825-0828*, or Δ*Gmet0825-0828*Δ*Gmet0908-0910* of *G. metallireducens* with *G. sulfurreducens* Δ*hybL*Δ*fdnG* (Fig. S6). Compared with that of the coculture with the WT of *G. metallireducens-G. sulfurreducens* Δ*hybL*Δ*fdnG*, the mRNA levels of 808 genes of *G. metallireducens* were elevated and 589 genes of *G. metallireducens* were decreased in the coculture with Δ*Gmet0825-0828* of *G. metallireducens-G. sulfurreducens* Δ*hybL*Δ*fdnG* (Fig. S7A and C; Table S7). These included eight upregulated *c*-Cyt genes and 29 downregulated *c*-Cyt genes (Table S7). The mRNA levels of 93 genes of *G. metallireducens* were elevated and 146 genes of *G. metallireducens* were decreased in the coculture with Δ*Gmet0825-0828*Δ*Gmet0908-0910* of *G. metallireducens-G. sulfurreducens* Δ*hybL*Δ*fdnG*, as compared with that in the coculture with the WT of *G. metallireducens-G. sulfurreducens* Δ*hybLL*Δ*fdnG* (Fig. S7B and C; Table S8). These included 13 upregulated *c*-Cyt genes and 15 downregulated *c*-Cyt genes (Table S8). Notably, *Gmet0911-0913* were among the genes whose mRNA levels increased 1.47- to 1.65-fold in the coculture with Δ*Gmet0825-0828*Δ*Gmet0908-0910* of *G. metallireducens-G. sulfurreducens* Δ*hybL*Δ*fdnG*, as compared with that with the WT of *G. metallireducens-G. sulfurreducens* Δ*hybL*Δ*fdnG* (Fig. 6A; Table S9). The mRNA levels of *Gmet0911-0913* in the coculture with Δ*Gmet0825-0828* of *G. metallireducens-G. sulfurreducens* Δ*hybL*Δ*fdnG*, however, were 25%–42% of that in the coculture with the WT of *G. metallireducens-G. sulfurreducens* Δ*hybL*Δ*fdnG* (Fig. 6A; Table S9). The mRNA levels of housekeeping gene *dnaK* of *G. metallireducens* remained constant in all the cocultures tested (Fig. 6A; Table S9).

To verify the transcriptomic results above, the polyclonal antibodies specific for Gmet0913 or DnaK were generated (Fig. S8). The protein levels of Gmet0913 or DnaK were compared with the antibodies in WT, Δ*Gmet0825-0828*, Δ*Gmet0908-0910*, and Δ*Gmet0825-0828*Δ*Gmet0908-0910* of *G. metallireducens* at 24 hours after reduction of Fe(III)-citrate, 10 days after reduction of ferrihydrite and 14 days after the first generation of their cocultures with *G. sulfurreducens* Δ*hybL*Δ*fdnG*. The results consistently showed that the protein levels of Gmet0913 in Δ*Gmet0825-0828*Δ*Gmet0908-0910* appeared higher than those in the WT, Δ*Gmet0825-0828*, and Δ*Gmet0908-0910* under the conditions tested. The levels of Gmet0913 in the WT appeared to be slightly higher than those in Δ*Gmet0825-0828* but appeared similar to that in Δ*Gmet0908-0910*. The protein levels of DnaK were constant in all strains tested (Fig. 6B).

## DISCUSSION

Previous results showed that deletion or modification of *pilA-N* of *G. metallireducens* did not impact the bacterial ability to reduce Fe(III)-citrate but diminished the bacterial ability to reduce Fe(III)-oxides and anodes and to form cocultures (8, 29, 30). Deletion of *Gmet0910*, *Gmet0912*, or *Gmet0913* negatively impacted the bacterial ability to form cocultures (10). Our results were consistent with these previous results.

Previous results also showed that deletion of *Gmet0910*, *Gmet0912*, or *Gmet0913* of *G. metallireducens* had little impact on the bacterial ability to reduce Fe(III)-citrate and Fe(III)-oxides (28). Our results, however, showed that deletion of *Gmet0908-0910* decreased the bacterial ability to reduce ferrihydrite, while deletion of *Gmet0911-0913* diminished the bacterial ability to reduce Fe(III)-citrate and ferrihydrite*.* This apparent discrepancy between our current and previous results is probably attributed to different bacterial mutants used in these studies. Previous investigation used single-gene-deletion mutants (28), while this investigation used single-gene-cluster-deletion mutants. Our results also showed that deletion of *Gmet0825-0828* had no impact on the bacterial ability to reduce Fe(III)-citrate but substantially decreased the bacterial ability to reduce ferrihydrite. Compared with that with empty vector, complementation of the single-gene-cluster-deletion mutants with their respective gene clusters fully improved the ability of the mutant to reduce Fe(III)-citrate but only partially improved the ability of the mutants to reduce ferrihydrite. The reasons for the partial complementation observed in this investigation are currently unknown.

In solution, multiple species of Fe(III)-citrate exist (33, 34). Their molecular masses are larger than 600 daltons that is the molecular mass cut-off for water-soluble molecules to pass through the outer membrane freely (33–35). Thus, bacterial reduction of Fe(III)-citrate occurred extracellularly. Involvement of *Gmet0911-0913* in extracellular reduction of Fe(III)-citrate not only was consistent with previous results showing the crucial roles of different protein porin-cytochrome complexes in the extracellular reduction of Fe(III)-citrate (17, 25, 26, 36) but also might contribute our failure of constructing the mutant without *Gmet0825-0828*, *Gmet0908-0910*, and *Gmet0911-0913* as the procedure for selecting the mutant was conducted under a Fe(III)-citrate-respiring condition. Deletion of *Gmet0825-0828*, *Gmet0908-0910*, and *Gmet0911-0913* probably rendered the mutant unable to grow via extracellular respiration of Fe(III)-citrate, which suggests the essential role of these *pcc* gene clusters in the EET of *G. metallireducens*.

Furthermore, our result showed for the first time involvements of *Gmet0825-0828*, *Gmet0908-0910*, and *Gmet0911-0913* in the extracellular reduction of anodes and involvement of *Gmet0825-0828* in the formation of cocultures between *G. metallireducens* and *M. barkeri* or the WT or *G. sulfurreducens* Δ*hybL*Δ*fdnG*. Involvement of these *pcc* gene clusters in extracellular reduction of ferrihydrite demonstrates their overlapping roles in ferrihydrite reduction by *G. metallireducens*. The overlapping EET functions of these *pcc* gene cluster could help bacterial survival when one of the *pcc* gene clusters inactivated by mutations.

Although they were involved in the extracellular reduction of Fe(III)-citrate, ferrihydrite, and/or anodes and coculture formation, the contributing roles of *Gmet0825-0828*, *Gmet0908-0910*, and *Gmet0911-0913* to these EET reactions varied substantially. Compared with that of *Gmet0908-0910* and *Gmet0911-0913*, deletion of *Gmet0825-0828* impacted more negatively on the extracellular reduction of ferrihydrite and cocultures between *G. metallireducens* and *M. barkeri* or the WT or *G. sulfurreducens* Δ*hybL*Δ*fdnG*. It should be noted that deletion of *Gmet0825-0828* impacted expressions of many other genes. Thus, its negative impact on extracellular reduction of ferrihydrite and cocultures between *G. metallireducens* and *M. barkeri* or the WT or *G. sulfurreducens* Δ*hybL*Δ*fdnG* might be attributed to the direct loss of this *pcc* gene cluster and/or altered expression of other genes. Moreover, failure of Δ*Gmet0825-0828* to reduce anodes and to form any stable coculture with either *M*. *barkeri* or the WT or *G. sulfurreducens* Δ*hybL*Δ*fdnG* clearly demonstrated the indispensable roles of *Gmet0825-0828* in extracellular reduction of anodes and DIET from *G. metallireducens* to *M*. *barkeri* as well as the WT and Δ*hybL*Δ*fdnG* of *G. sulfurreducens*. It should be noted that the mRNA levels of *Gmet0911-0913* and the protein level of Gmet0913 were also lower than those in the WT, which might contribute to the phenotypes of Δ*Gmet0825-0828* in the EET observed in this study. Similarly, *Gmet0908-0910* is also indispensable for extracellular reduction of anodes. Compared with *Gmet0911-0913*, *Gmet0908-0910* played no apparent role in Fe(III)-citrate reduction, a less dominant role in ferrihydrite reduction, but more dominant roles in DIET from *G. metallireducens* to *M*. *barkeri* and the WT and *G. sulfurreducens* Δ*hybL*Δ*fdnG*. *Gmet0911-0913* was involved in reductions of Fe(III)-citrate, ferrihydrite, and anodes, but its roles in the cocultures were trivial. Collectively, these results clearly showed the distinct roles of these *pcc* gene clusters in the different EET reactions of *G. metallireducens*. The distinct EET functions of its *pcc* gene clusters enable *G. metallireducens* to use different electron acceptors more efficiently during EET reactions.

Most importantly, our results also showed for the first time that the ability of the double-gene-cluster-deletion-mutantΔ*Gmet0825-0828*Δ*Gmet0908-0910* to reduce ferrihydrite and anodes and to mediate DIET from *G. metallireducens* to *M*. *barkeri* and the WT and *G. sulfurreducens* Δ*hybL*Δ*fdnG* increased significantly, as compared with that of the single-gene-cluster-deletion mutants of Δ*Gmet0825-0828* and Δ*Gmet0908-0910*. Our results further revealed that the improved EET capabilities of Δ*Gmet0825-0828*Δ*Gmet0908-0910* were attributed at least in part to the elevated mRNA levels of *Gmet0911-0913* and protein levels of *Gmet0913* and probably *Gmet0911* and *Gmet0912.* Thus, *G. metallireducens* might compensate for the loss of EET functions of *Gmet0825-0828* and *Gmet0908-0910* via at least in part the increased expression of *Gmet0911-0913*. The compensatory function of *Gmet0911-0913* in the EET of *G. metallireducens* is hypothesized to have substantially improved bacterial growth when both *Gmet0825-0828* and *Gmet0908-0910* were deleted. This discovered compensatory function differs significantly from the improved anode reduction of the mutant from *G. sulfurreducens* with only the *extABCD pcc* gene cluster (*extABCD*^+^) (24, 25). The improved EET capability of *extABCD*^+^ was only observed in anode reduction via the mechanisms other than increased expression of *extABCD*, such as improved biofilm formation on anodes and metabolic activity (24, 25). Additionally, Δ*Gmet0825-0828*, Δ*Gmet0908-0910*, and Δ*Gmet0911-0913* behaved differently from their counterparts in *G. sulfurreducens* in terms of Fe(III) and anode reductions. For example, Δ*Gmet0911-0913* showed diminished reduction of Fe(III)-citrate, while Δ*omaBombBomcB* and Δ*omaCombComcC* of *G. sulfurreducens* reduced Fe(III)-citrate normally (17, 25). These results indicated that the *pcc* homologs in *Geobacter* spp. might function differently.

Collectively, the results of this investigation demonstrated not only the essential but also the distinct, overlapping as well as compensatory roles of the *pcc* gene clusters in the EET of *G. metallireducens*. The distinct EET functions of the *pcc* gene clusters permit *G. metallireducens* to transfer electrons efficiently to the extracellular acceptors of different properties. The overlapping and compensatory EET functions enable *G. metallireducens* to mediate EET for bacterial growth even after two of its three gene clusters become defective by mutations. These results provide new insights into the robust roles of *pcc* gene clusters in bacterial EET.

## MATERIALS AND METHODS

### Microbial strains and cultivation conditions

*Geobacter metallireducens* GS-15 (ATCC 53774), *Geobacter sulfurreducens* PCA (ATCC 51573), and *Methanosarcina barkeri* MS (ATCC 51582) were purchased from American Type Culture Collection (Manassas, VA, USA) (Table S1). All microorganisms were cultured under anoxic condition (N_2_/CO_2_, 80%/20%, vol/vol) and maneuvered in a Coy anaerobic chamber (Coy Laboratory Products Inc., Grass Lake, MI, USA). *G. metallireducens* and *G. sulfurreducens* were cultured at 30°C in the bicarbonate-buffered media (2 g/L NaHCO_3_, 0.38 g/L KCl, 0.2 g/L NH_4_Cl, 0.069 g/L NaH_2_PO_4_·H_2_O, 0.04 g/L CaCl_2_·2H_2_O, 0.2 g/L MgSO_4_·7H_2_O, 1% [vol/vol] NB trace mineral mix, pH 6.8) with 20 mM acetate as the sole electron donor and 56 mM Fe(III)-citrate or 40 mM fumarate as the sole electron acceptor, respectively (17, 29, 31, 37). *M. barkeri* was grown at 37°C in the modified DSM 120 medium (2 g/L NaHCO_3_, 1 g/L NaCl, 0.35 g/L K_2_HPO_4_, 0.5 g/L NH_4_Cl, 0.002 g/L CaCl_2_·2H_2_O, 0.5 g/L MgSO_4_·7H_2_O, 0.1 g/L L-cysteine HCl, 0.04 g/L Na_2_S·9H_2_O, 0.2% [vol/vol] FeSO_4_·7H_2_O solution [0.1% wt/vol], 0.1% [vol/vol] SL-10 trace element solution, 0.1% [vol/vol] Wolin’s vitamin solution [10×], pH 6.8) with 30 mM acetate as the electron donor (8). Acetate, fumarate, and Fe(III)-citrate were purchased Sinopharm Chemical Reagent Co. Ltd. (Shanghai, China).

### Construction of mutants for *G. metallireducens* and *G. sulfurreducens*

The mutants without *pcc* gene clusters or *pilA-N* gene for *G. metallireducens* or without *hybL* and *fdnG* genes for *G. sulfurreducens* were constructed by following previously published protocols (17, 26, 29, 31, 38–40). Briefly, three fragments were generated through PCR amplification for the single gene deletion: 500 bp upstream fragment and 500 bp downstream fragment of the target gene and the spectinomycin resistance cassette flanked by the *loxP* site. These fragments and linearized plasmid pUC19 were joined with the In-Fusion HD Cloning Kit (Takara Biomedical Technology, Beijing, China) to generate a circle plasmid. After verification with DNA sequencing of inserts, the plasmids were cut with *ScaI* (NEB, Ipswich, MA, USA) and electroporated into the electrocompetent *Geobacter* cells. PCR and DNA sequencing of the deleted regions were used for verifying the constructed mutants. After verification, the spectinomycin resistance cassette was excised from the chromosome by expressing the Cre recombinase from plasmid pCM158 (29). Double gene deletion mutants were made by repeating the above steps. The verified mutants of *G. metallireducens* were complemented with their respective genes cloned into the plasmid pCM66 separately (41). *Escherichia coli* DH5α was used for cloning and purchased from Takara Biomedical Technology (Beijing, China) (42) (Table S1). The mutants of *G. metallireducens* were also transformed with empty vector pCM66 as controls. Kanamycin was used at a final concentration of 200 µg/mL in the complement assays. Tables S1 and S2 list all microbial strains and plasmids and oligonucleotide primers used in this investigation, respectively.

### Reductions of Fe(III)-citrate and ferrihydrite

The constructed mutants of *G. metallireducens* were tested for their ability to reduce Fe(III)-citrate and ferrihydrite. Ferrihydrite was prepared and characterized by following a protocol described previously (31, 36, 43). Briefly, ferrihydrite was synthesized by dropwise addition of 1 M NaOH to 500 mL of 0.2 M FeCl_3_ until pH 7.0 was reached. The suspension was centrifuged (30 min, 2,100 × *g*, 20°C), washed with doubly deionized water (ddH_2_O) prepared with a Milli-Q system (Millipore, Billerica, MA, USA), and freeze dried. X-ray diffraction with a Bruker AXS GmbH D8-Focus-Power Diffraction System (Bruker, Billerica, MA, USA), scanning electron microscopy with a HITACHI SU8010 microscope (Hitachi, Chiyoda, Japan), transmission electron microscopy with a Tecnai G20 TWIN microscope (Thermo Fisher Scientific, Waltham, MA, USA), and an ASAP 2460 Accelerated Surface Area & Porosity Analyzer (Micromeritics, Norcross, GA, USA) were used to characterize the synthesized ferrihydrite. The characterized ferrihydrite was stored at 4°C and used within 60 days. Reductions of Fe(III)-citrate and ferrihydrite were measured as described before (17, 26, 31).

### Growth on anodes

Growth of the WT and mutants for *G. metallireducens* on anodes (polished graphite plate of 3.2 cm^2^) as the sole electron acceptor was carried in microbial fuel cells (MFCs) of a single chamber with three electrodes (44). The current production of MFCs was monitored with a potentiostat (+300 mV versus Ag/AgCl) (CH Instruments Inc., Shanghai, China). At the end of each growth, the total amount of the proteins on the anode was measured with the Qubit Protein Assay Kit (Invitrogen Life Technologies, Carlsbad, CA, USA) and a Qubit Fluorometer. The biofilms formed on the anodes were visualized with a Leica TCS SP8 MP Multiphoton Confocal Microscope (Wetzlar, Germany) after being stained with the LIVE/DEAD BacLight Bacterial Viability Kits (Thermo Fisher Scientific China Co. Ltd., Shanghai, China).

### Cocultures

Prior to coculture, the WT and mutants of *G. metallireducens* were grown in the bicarbonate-buffered medium of 56 mM Fe(III)-citrate in which acetate was replaced with 20 mM ethanol (7, 8). The preadapted strains of *G. metallireducens* were then cocultured with *M. barkeri* or the WT or Δ*hybL*Δ*fdnG* of *G. sulfurreducens*. The cocultures between the strains of *G. metallireducens* and *M. barkeri* were conducted at 37°C in the modified DSM 120 medium in which acetate was replaced with 20 mM ethanol (8), while the cocultures between the strains of *G. metallireducens* and the strains of *G. sulfurreducens* were conducted at 30°C in the bicarbonate-buffered medium of 20 mM ethanol and 40 mM fumarate (7).

During the cocultures, the concentrations of ethanol and organic acids were measured with a LC-20A Shimadzu high-performance liquid chromatography (HPLC) system that also contained a SPD-M20A UV detector, a RID-20A high-sensitivity refractive index detector (Shimadzu, Kyoto, Japan), and an Aminex HPX-87H Ion Exclusion column (300 × 7.8 mm) (Bio-Rad Laboratories Inc., California, USA). The ethanol and organic acids were purchased from Sinopharm Chemical Reagent Co. Ltd. The methane produced during the cocultures between the strains of *G. metallireducens* and *M. barkeri* was measured with a GC-2014 Shimadzu gas chromatograph system with as a flame ionization detector. Methane (99.99% purity) was purchased from Wuhan Steel Group Commercial Gasses Co. Ltd. (Wuhan, China). The copies of microbial 16S rRNA genes were measured with a QuantStudio3 (Thermo Fisher Scientific China Co. Ltd.) via quantitative PCR (qPCR) with TB Green Premix Ex Taq II (Takara Biomedical Technology, Beijing, China). The microbial genomic DNAs of cocultures were isolated with the TIANamp Bacteria DNA Kit (TIANGEN, Beijing, China). The granules formed after cocultures were examined with a Leica TCS SP8 MP multiphoton confocal microscope after FISH. The primers used in qPCR and FISH are listed in Table S2.

### RNA extraction and transcriptomic analysis

Microbial cells of cocultures between the strains of *G. metallireducens* and *G. sulfurreducens* Δ*hybL*Δ*fdnG* were harvested at 4°C by centrifugation (5,000 × *g*, 15 min) at a predetermined time point and then frozen with liquid nitrogen. RNA was extracted using a Magen HiPure Bacterial RNA Kit (Magen Biotechnology Co. Ltd., Guangdong, China) and verified on an agarose gel. rRNA was removed with the Epicentre Ribo-Zero rRNA Removal Kit (Illumina, San Diego, CA, USA). The cDNA libraries were constructed using a NEBNext Ultra II Directional RNA Library Prep Kit (Illumina) and then sequenced using an Illumina NovaSeq PE150 platform at the Guangdong Magigene Biotechnology Co. Ltd., China (Guangdong, China).

Trimmomatic was used to trim the raw reads (45). Bowtie2 combined with RefSeq and Rfam 14 was used to further remove rRNA reads from trimmed reads (46–48). The non-rRNA reads were compared with the genomes of *G. metallireducens* (NC_007517.1) and *G. sulfurreducens* (NC_002939.5) with Bowtie2. The transcripts per million (TPM) method was used to normalize the gene abundances in their respective genomes (49). The edgeR was used to analyze differential expressions of genes (50). RStudio was used to visualize the results of principal component analysis of Bray-Curtis distance and MD plots methods.

### Antibody productions and immunoblot analyses

The Gme0913- or DnaK-specific antibodies were made by Proteintech Group, Inc. (Wuhan, China), and were characterized by following the protocols described before (14, 51). The polypeptides used for making these polyclonal antibodies are listed in Table S3. The cells of WT and mutants of *G. metallireducens* that were in reductions of Fe(III)-citrate or ferrihydrite or cocultured with *G. sulfurreducens* Δ*hybL*Δ*fdnG* were harvested at 4°C by centrifugation (5,000 × *g*, 15 min) at predetermined time points. The cells were washed with ice-cold Tris-buffered saline (TBS; pH 7.6) three times and were then re-suspended in TBS to the same optical density at 600 nm (OD_600_). Equal-amount cells were lysed with the sodium dodecyl sulfate-polyacrylamide gel electrophoresis (SDS-PAGE)loading buffer. The cell lysates were loaded on 10% SDS-polyacrylamide gels and then separated by SDS-PAGE. The separated proteins were transferred to polyvinylidene flouride (PVDF) membranes (Thermo Fisher Scientific China Co. Ltd.). The membranes were then analyzed with Gme0913- or DnaK-specific antibodies. Goat anti-rabbit IgG-HRP and pageruler prestained protein ladder were purchased from TransGen Biotech (Beijing, China) and Thermo Fisher Scientific China Co. Ltd,. respectively. The interactions between antibodies and their respective target proteins were visualized with the super sensitive ECL luminescence reagent (Meilunbio, Dalian, China) and detected with Azure C300 (Azure Biosystems Inc, Dublin, CA, USA).

### Statistical analyses

All values are expressed as means ± standard deviations. Student’s *t* test was used for comparing groups.

## Data Availability

The transcriptomic data from this investigation were submitted to NCBI BioProject Database with BioProject ID PRJNA1128387.

## SUPPLEMENTAL MATERIAL

Supplemental figures mbio.00580-24-s0001.docx Figures S1 to S8.

Supplemental tables mbio.00580-24-s0002.xlsx Tables S1 to S9.

ASM does not own the copyrights to Supplemental Material that may be linked to, or accessed through, an article. The authors have granted ASM a non-exclusive, world-wide license to publish the Supplemental Material files. Please contact the corresponding author directly for reuse.
