# Supplementary material for: The robustness of porin-cytochrome gene clusters from Geobacter metallireducens in extracellular electron transfer
Source: mBio. 2024 Aug 2;15(9):e00580-24. doi: 10.1128/mbio.00580-24 (PMC11389400; doi:10.1128/mbio.00580-24)
Supplement: Supplemental figures — Figures S1 to S8. [file mbio.00580-24-s0001.docx]

## The robustness of porin-cytochrome gene clusters from *Geobacter metallireducens* in extracellular electron transfer

Shiyan Zhuo^1^, Yongguang Jiang^1^, Lei Qi^2^, Yidan Hu^1^, Zhou Jiang^1^, Yiran Dong^1,3,4,5^, Liang Shi^1,3,4,5*^

^1^ Department of Biological Sciences and Technology, School of Environmental Studies, China University of Geosciences, Wuhan, China

^2^ State Key Laboratory of Microbial Resources, Institute of Microbiology, Chinese Academy of Sciences, Beijing, China

^3^ State Key Laboratory of Biogeology and Environmental Geology, China University of Geosciences, Wuhan, China

^4^State Environmental Protection Key Laboratory of Source Apportionment and Control of Aquatic Pollution, Ministry of Ecology and Environment, China University of Geosciences, Wuhan, China

^5^Hubei Key Laboratory of Yangtze Catchment Environmental Aquatic Science, China University of Geosciences, Wuhan, China

9 pages

8 figures.


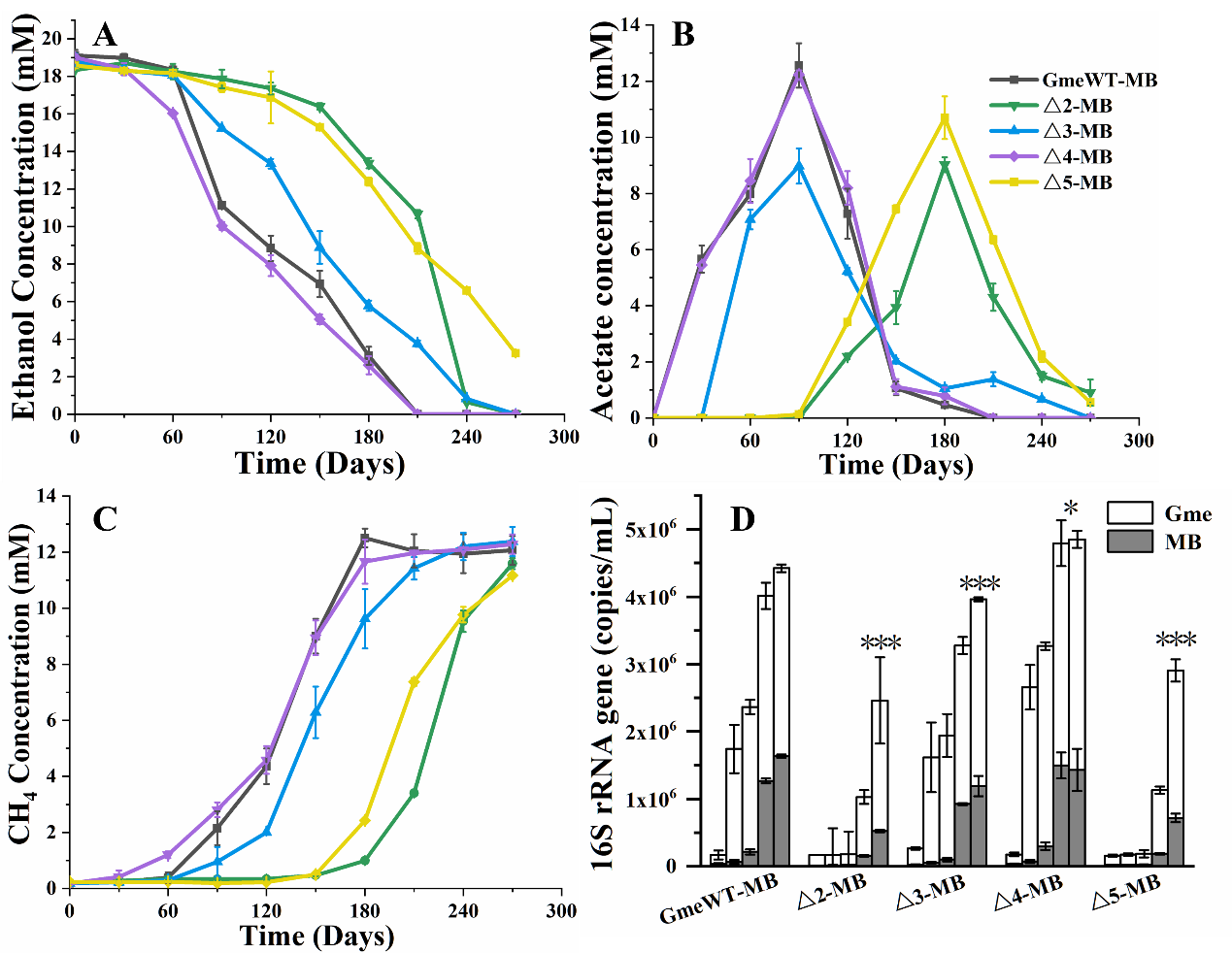


**Figure S1.** The second generation of cocultures between the wild-type (WT) and gene-deletion mutants of *G. metallireducens* (Gme) and *Methanosarcina barkeri* (MB). (A) Ethanol metabolism. (B) Acetate metabolism. (C) Methane production. (D) The copies of combined microbial 16S rRNA genes. All results are reported as mean and standard error of the mean (n = 3). For points with no error bar, the error was smaller than the size of the symbol. In (D), samples were collected at 0, 60, 120, 180 and 240 days after cocultures. Student’s *t* test was used for comparing the maximum copies of combined microbial 16S rRNA genes. *, *p* ≤ 0.05; ***, *p* ≤ 0.001. WT, wild-type of *G. metallireducens*; Δ2, Δ*Gmet0908-0910*; Δ3, Δ*Gmet0911-0913*; Δ4, Δ*Gmet0825-0828*Δ*Gmet0908-0910*; Δ5, Δ*pilA-*N.


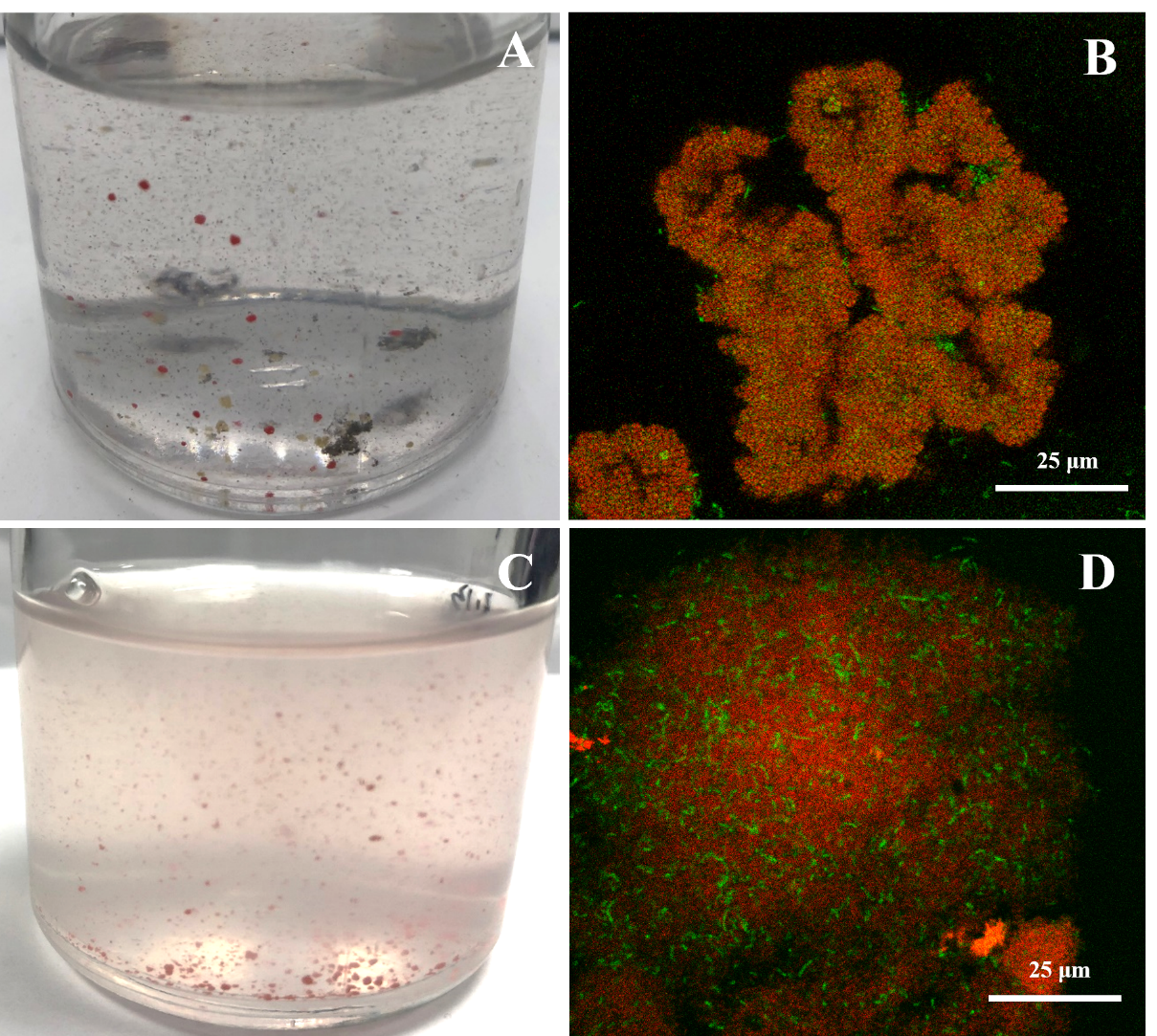


**Figure S2.** Granule formation and fluorescence *in situ* hybridization (FISH) analyses of cocultures between *Geobacter metallireducens* and *Methanosarcina barkeri* or *Geobacter sulfurreducens*. (A) Granules formed at 180 days after second generation of coculture between *G. metallireducens* and *M. barkeri*. (B) FISH analyses of the granules in (A). Green, *G. metallireducens*; red, *M. barkeri*. (C) Granules formed at 21 days after second generation of coculture between *G. metallireducens* and *G. sulfurreducens*. (D) FISH analyses of the granules in (C). Green, *G. metallireducens*; red, *G. sulfurreducens*.

**
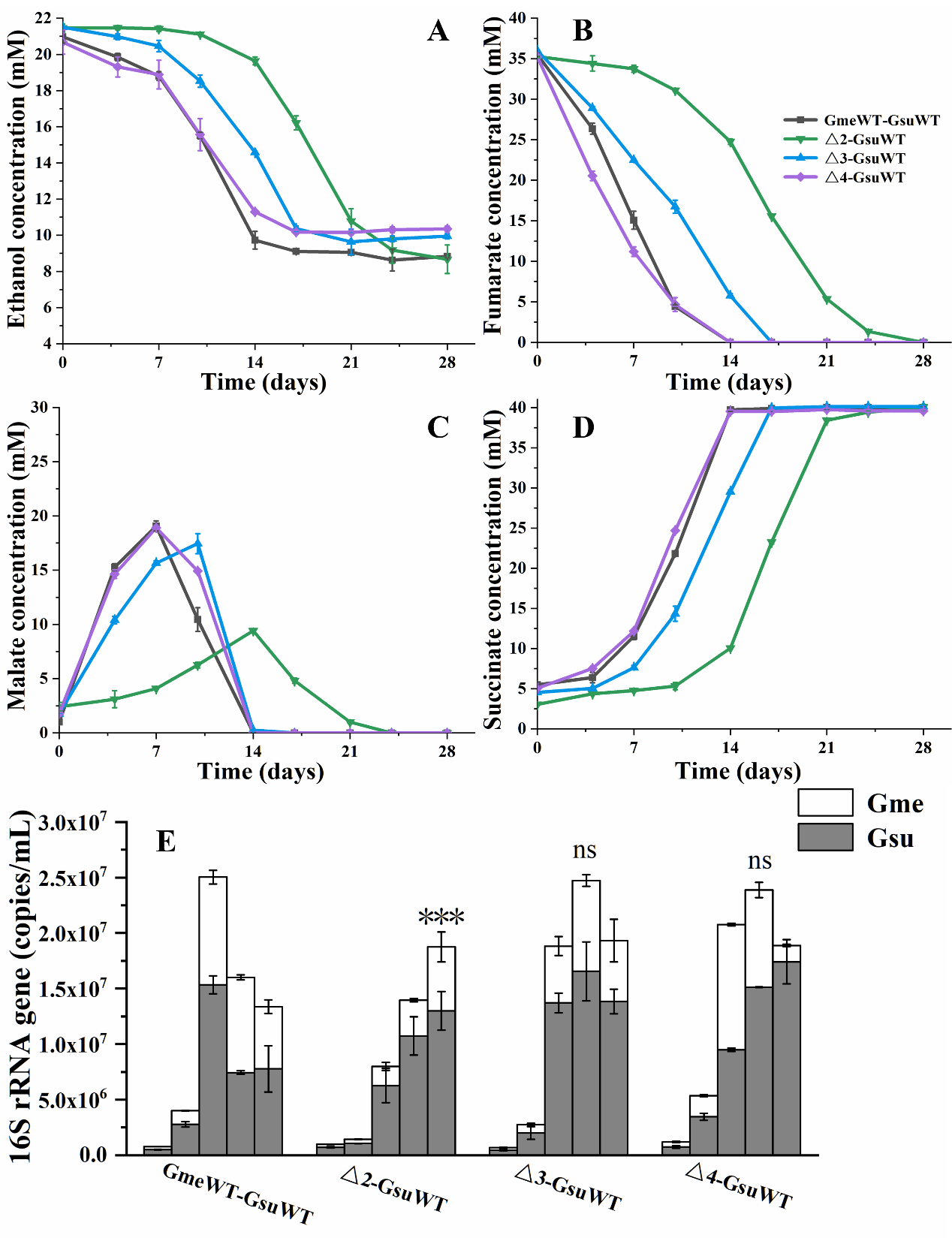
Figure S3**. The second generation of cocultures between wild-type (WT) and gene-deletion mutants of *Geobacter metallireducens* (Gme) and *Geobacter sulfurreducens* (Gsu)*.* (A) Ethanol metabolism. (B) Fumarate metabolism. (C) Malate metabolism. (D) Succinate production. (E) The copies of combined bacterial 16S rRNA genes. All results are reported as mean and standard error of the mean (n = 3). For points with no error bar, the error was smaller than the size of the symbol. In (E), samples were collected at 0, 7, 14, 21 and 28 days after cocultures. Student’s *t* test was used for comparing the maximum copies of combined bacterial 16S rRNA genes. ns, *p* > 0.05; ***, *p* ≤ 0.001. WT, wild-type of *G. metallireducens*; Δ2, Δ*Gmet0908-0910*; Δ3, Δ*Gmet0911-0913*; Δ4, △*Gmet0825-0828*Δ*Gmet0908-0910*.

**
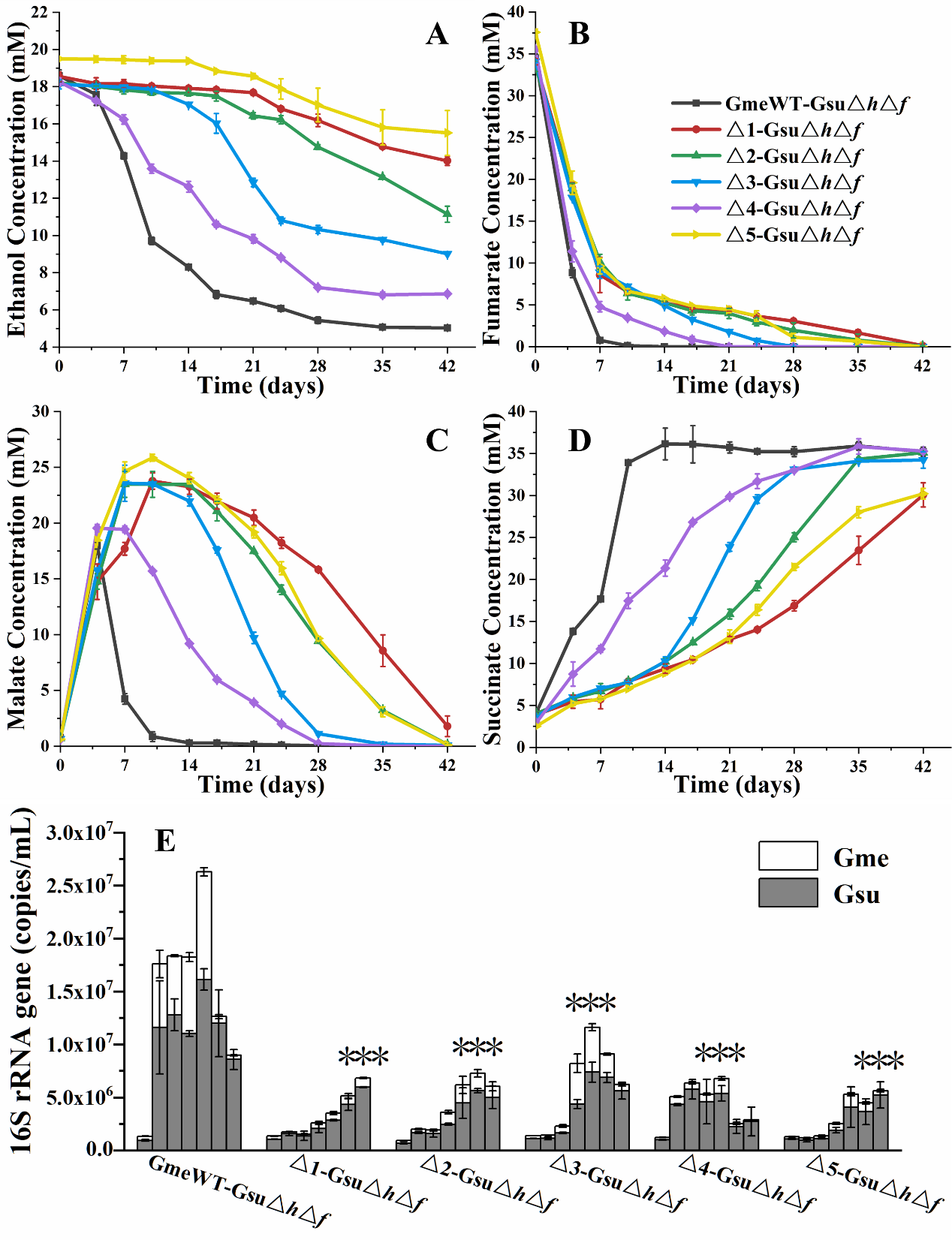
Figure S4.** The first generation of cocultures between wild-type (WT) and gene-deletion mutants of *Geobacter metallireducens* (Gme) and *Geobacter sulfurreducens* Δ*hybL*Δ*fdnG* (GsuΔ*h*Δ*f*)*.* (A) Ethanol metabolism. (B) Fumarate metabolism. (C) Malate metabolism. (D) Succinate production. (E) The copies of combined 16S rRNA genes of *G. metallireducens* and *G. sulfurreducens*. All results are reported as mean and standard error of the mean (n = 3). For points with no error bar, the error was smaller than the size of the symbol. In (E), samples were collected at 0, 7, 14, 21, 28, 35 and 42 days after cocultures. Student’s *t* test was used for comparing the maximum copies of combined bacterial 16S rRNA genes. ***, *p* ≤ 0.001. WT, wild-type of *G. metallireducens*; Δ1, Δ*Gmet0825-0828*; Δ2, Δ*Gmet0908-0910*; Δ3, Δ*Gmet0911-0913*; Δ4, Δ*Gmet0825-0828*Δ*Gmet0908-0910*.

**
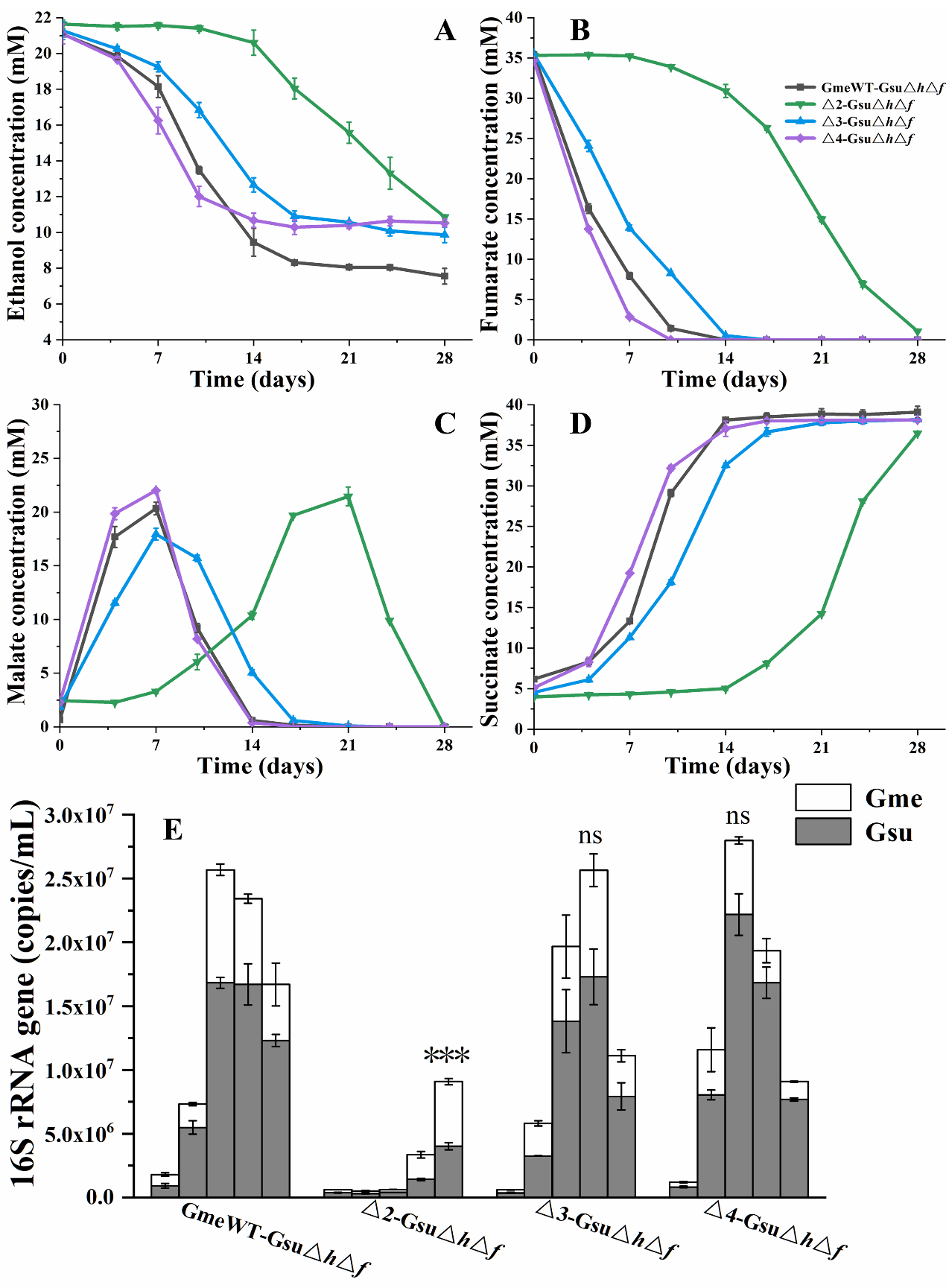
Figure S5.** The second generation of cocultures between wild-type (WT) and gene-deletion mutants of *Geobacter metallireducens* (Gme) and *Geobacter sulfurreducens* Δ*hybL*Δ*fdnG* (GsuΔ*h*Δ*f*)*.* (A) Ethanol metabolism. (B) Fumarate metabolism. (C) Malate metabolism. (D) Succinate production. (E) The copies of combined 16S rRNA genes of *G. metallireducens* and *G. sulfurreducens*. All results are reported as mean and standard error of the mean (n = 3). For points with no error bar, the error was smaller than the size of the symbol. In (E), samples were collected at 0, 7, 14, 21 and 28 days after cocultures. Student’s *t* test was used for comparing the maximum copies of combined bacterial 16S rRNA genes. ns, *p* > 0.05; ***, *p* ≤ 0.001. WT, wild-type of *G. metallireducens*; Δ2, Δ*Gmet0908-0910*; Δ3, Δ*Gmet0911-0913*; Δ4, Δ*Gmet0825-0828*Δ*Gmet0908-0910*.


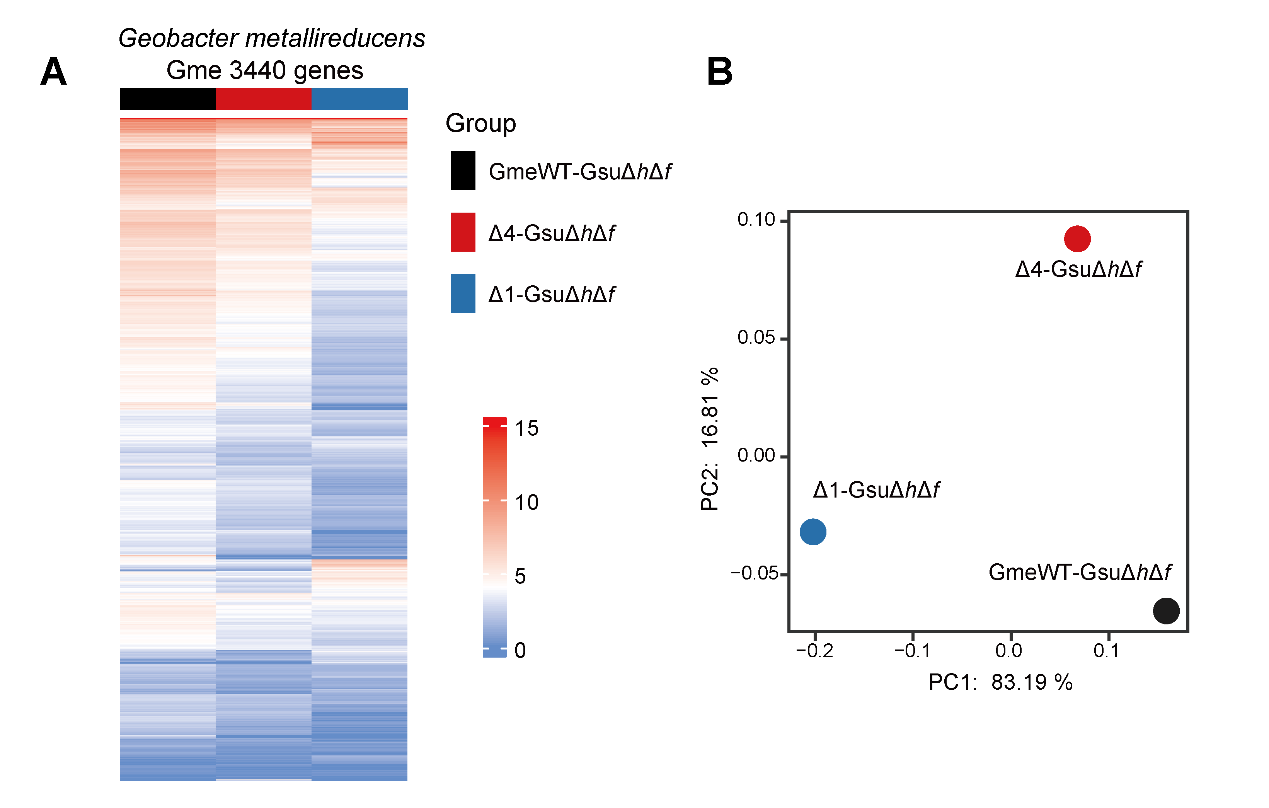


**Figure S6.** Transcriptomic comparison of the first generation of cocultures of the wild-type of (WT) and gene-deletion mutants of *Geobacter metallireducens* (Gme) and *Geobacter sulfurreducens* Δ*hybL*Δ*fdnG* (GsuΔ*h*Δ*f*) at 14 days after the cocultures. (A) The heatmaps of all genes identified in the cocultures. (B) Multidimensional scaling plots of the gene expression of the cocultures. Δ1, Δ*Gmet0825-0828*; Δ4, Δ*Gmet0825-0828*Δ*Gmet0908--0910*.


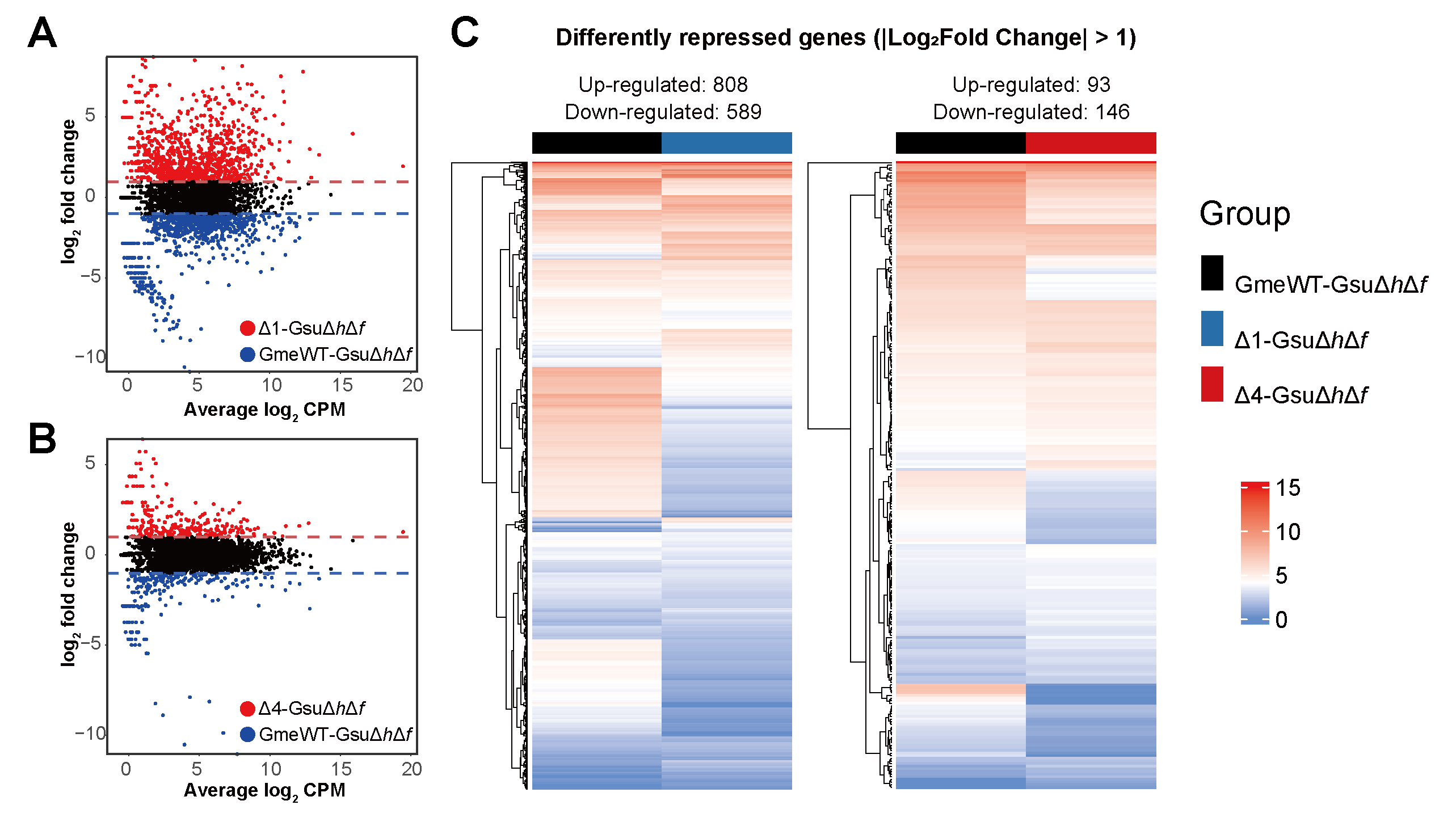


**Figure S7.** Differential comparison of the genes expressed in the first generation of cocultures of the wild-type of (WT) and gene-deletion mutants of *Geobacter metallireducens* (Gme) and *Geobacter sulfurreducens* Δ*hybL*Δ*fdnG* (GsuΔ*h*Δ*f*) at 14 days after the cocultures. (A) Mean-difference (MD) plots for the comparison between the cocultures of Δ*Gmet0825-0828*-GsuΔ*h*Δ*f* and the control GmeWT-GsuΔ*h*Δ*f*. (B) MD plots for the comparison between the cocultures of Δ*Gmet0825-0828*Δ*Gmet0908--0910*-GsuΔ*h*Δ*f* and the control GmeWT-GsuΔ*h*Δ*f*. (C) The heatmaps of differentially expressed genes in the cocultures of GmeWT-GsuΔ*h*Δ*f*, Δ*Gmet0825-0828*-GsuΔ*h*Δ*f* and Δ*Gmet0825-0828*Δ*Gmet0908--0910*-GsuΔ*h*Δ*f*. Δ1, Δ*Gmet0825-0828*; Δ4, Δ*Gmet0825-0828*Δ*Gmet0908--0910*.


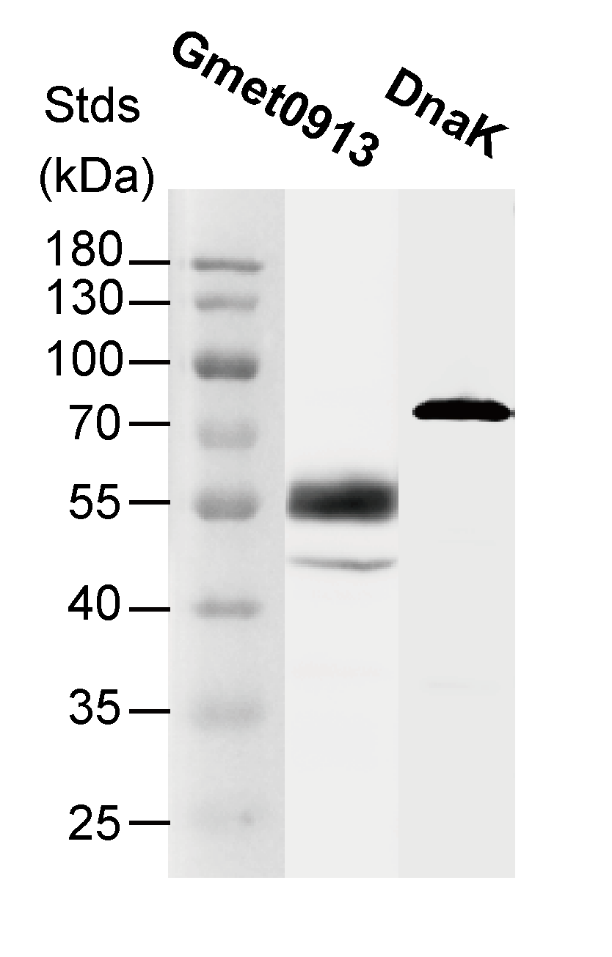


**Figure S8.** Immunoblot characterization of the antibodies specific for Gmet0913 or DnaK. The migration positions of standard proteins (Stds) in kilodaltons (kDa) are shown on the left.
